# S-ICD Implantation "Tips and Tricks"

**DOI:** 10.31083/j.rcm2407195

**Published:** 2023-07-10

**Authors:** Szymon Budrejko, Maciej Kempa, Andrzej Przybylski

**Affiliations:** ^1^Department of Cardiology and Electrotherapy, Medical University of Gdansk, 80-210 Gdansk, Poland; ^2^1st Department of Cardiology with the Acute Coronary Syndromes Subdivision, Clinical Provincial Hospital No. 2, 35-310 Rzeszow, Poland; ^3^Medical College of Rzeszow University, 35-310 Rzeszow, Poland

**Keywords:** implantable cardioverter-defibrillator, subcutaneous implantable cardioverter-defibrillator, sudden cardiac death

## Abstract

An implantable cardioverter-defibrillator (ICD) was developed to provide protection against sudden cardiac death. Despite being effective in terminating ventricular arrhythmias, traditional transvenous ICDs appeared over time to have certain limitations related to the need for vascular access and the presence of foreign material inside the circulatory system (namely lead failure and infections). A subcutaneous implantable cardioverter-defibrillator (S-ICD) was developed to overcome those limitations and to provide prevention against sudden cardiac death from outside the cardiovascular system. Utilization of that modern method of treatment is constantly increasing worldwide, and new centers incorporate implantation of that system in their portfolio. This review aims to present the most relevant issues related to S-ICD implantation procedure, based on experience of the authors and an extensive literature search.

## 1. Introduction

Implantable cardioverter-defibrillator (ICD) is an established method used for prevention of sudden cardiac death (SCD). Its role in the secondary prevention of SCD in cardiac arrest survivors is indisputable, and also the primary prevention in patients with low left ventricular ejection fraction widely employs ICDs [1]. The worldwide career of transvenous ICDs (TV-ICDs) relies on their high efficacy, a commonly known implantation technique and a relatively long history. But a TV-ICD has its Achilles heel - the lead. Possible lead-related complications include: lead failure, lead-dependent infective endocarditis, cardiac perforation, and venous thrombosis. Despite the growing worldwide experience in transvenous lead removal, those issues may and do negatively influence the prognosis of ICD recipients [2]. For that reason, a subcutaneous implantable cardioverter-defibrillator (S-ICD) system has been invented and successfully introduced into clinical practice [3]. Being located exclusively in the subcutaneous tissue of the thorax, and having no direct communication with the cardiovascular system, it provides antiarrhythmic therapy without the risk of vascular and/or lead-related complications typical for TV-ICDs [4, 5]. The ongoing dissemination of that modern technology requires cardiac electrophysiologists to modify their habits, as well as to learn new concepts and implantation techniques, because the implantation procedure is for obvious reasons different from what they knew for TV-ICDs. In this review, based on extensive literature search and on our own experience, we intend to provide an in-depth description of S-ICD implantation procedure, and various tips and tricks, including some measures to avoid possible complications specific to that modality of treatment.

## 2. General Description of the S-ICD System

The S-ICD system, similarly to a TV-ICD, consists of a device can and a lead, but the lead is implanted in the subcutaneous tissue and has no contact with the venous system and the heart itself. The can is up to two times larger than in TV-ICDs, it measures approximately 8.3 by 6.9 cm and has a thickness of 1.3 cm, which results in a volume of 60 cubic centimeters, and it weighs 130 g. Therefore the patient’s body build has to be taken into account, as the implantation procedure of that relatively large device may be more challenging in very thin and small patients, including children. In those patients, the can may be more prominent on the lateral wall of the chest and might even have a tendency for rocking movements. The likelihood of local complications (lead or can erosion or decubitus) might be also higher in such cases [6]. Despite those concerns, S-ICDs have been successfully implanted in children over 8 years of age and over 38 kg of body mass [7], or with the body mass index (BMI) over 20 kg/$m^{2}$ [6, 8]. The results of a recent meta-analysis may favor S-ICD over TV-ICD in the young population, but a trend towards a higher risk of pocket complications [9] raises concern and underlines the need for a meticulous surgical technique.

Arrhythmia detection of an S-ICD relies on the analysis of subcutaneously recorded electrocardiography (ECG)-like signals. As such, they have some characteristics of a surface ECG (vulnerability to record noise and muscular artifacts) and rely on the analysis of morphology of the signals, as opposed to the intracardiac signals recorded by TV-ICDs, being more binary in nature. The S-ICD system applies a detection algorithm to one of the three possible signal vectors. Those vectors are recorded between any two of the three available subcutaneous poles, being the can itself and two sensing rings on the lead. One ring is located close to the tip, and the other one is —14 cm proximally, just above the anchoring sleeve (the lead itself has 45 cm of length, only one length is available and has to fit all patients). The sensing vector is selected automatically by the device during optimization procedure after implantation (preceded by an automated analysis of all the signals in two body positions). It can be manually changed, which is not recommended by the manufacturer, unless necessary. The device is capable of delivering shocks of the energy up to 80 Joules. A form of post-shock pacing is available at 50 bpm for 30 seconds. It is not programmable (it can only be switched on or off), and it is executed by impulses between the coil and the can of the device (a concept similar to transcutaneous pacing, without direct physical contact with the heart). The system is magnetic resonance imaging (MRI) conditional [10].

## 3. Qualification and Preoperative ECG Screening

Several issues have to be deliberated upon if we weigh implantation of an S-ICD system against the typical TV-ICD. Two main contraindications for an S-ICD are: the need for antibradycardia pacing or cardiac resynchronization therapy (although in our opinion some individual exceptions are possible and will be discussed below) and the possible need for antitachycardia pacing (ATP) for ventricular tachycardia (VT), especially in patients with slow and stable VTs. Some clinical conditions speak in favor of S-ICD, namely the young age of a patient (or in other words — long life expectancy), a history of lead-related complications (repetitive lead failure), prior infective complications (either lead-related or pocket-related), chronic conditions that increase the risk of ICD infection (chronic local or systemic infections, immunosuppressive therapy, chronic steorid therapy, hemodialysis), and problematic vascular access (venous thrombosis, atypical venous anatomy, congenital heart disease). According to the current European Society of Cardiology (ESC) guidelines, an S-ICD should be considered as an alternative to a TV-ICD in patients with an ICD indication when pacing therapy for bradycardia, cardiac resynchronization therapy, or ATP is not needed. That is a class IIa indication with level of evidence B [1]. The medical conditions listed above may theoretically justify the choice of S-ICD instead of TV-ICD, but rules for reimbursement are country-specific and may vary, therefore precise legal requirements and local regulations have to be carefully considered and obeyed in each case.

**Fig. 1.**
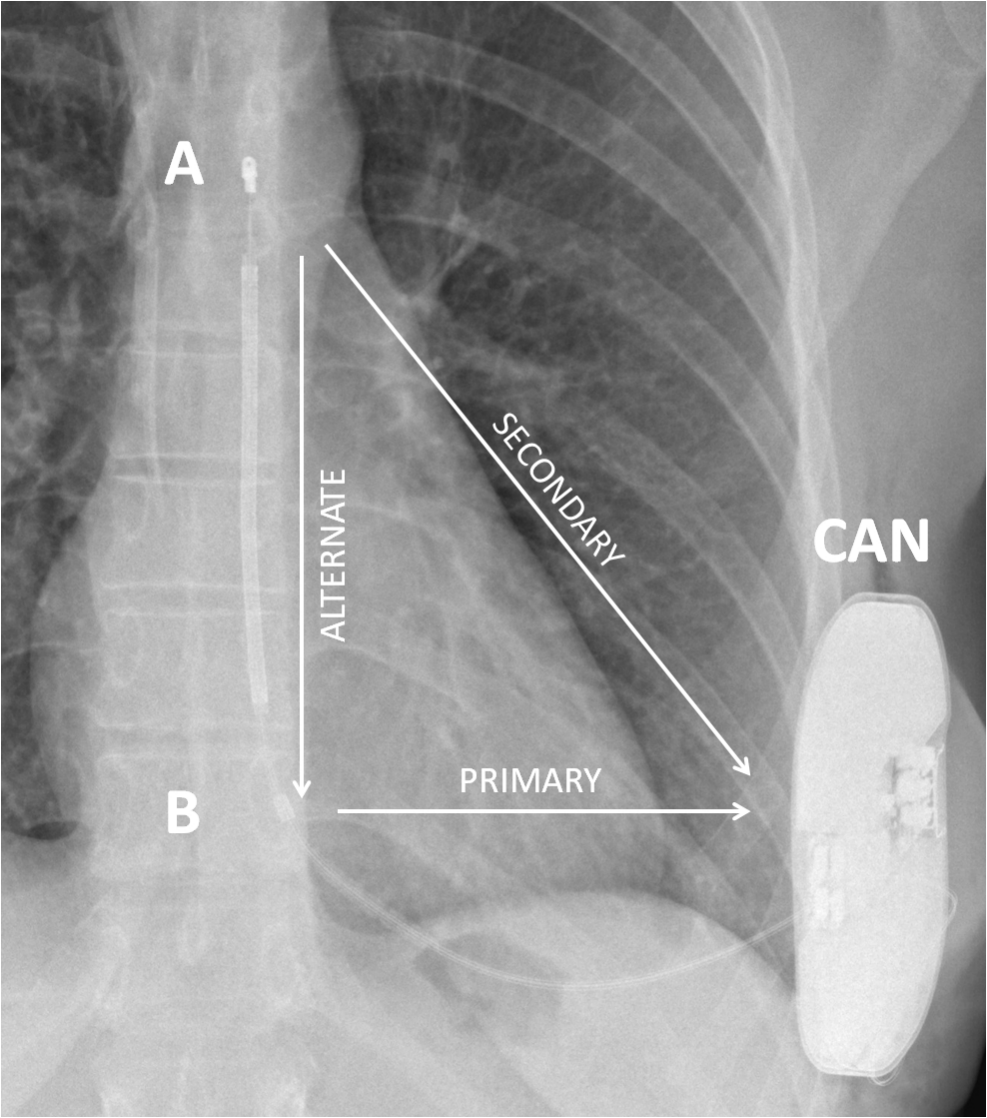
**Schematic representation of the three sensing vectors available in the S-ICD system**. S-ICD, subcutaneous implantable cardioverter-defibrillator.

The next step of a decision-making path incorporates the so-called ECG screening. As mentioned above, the device has three sensing poles (two on the lead, one is the can itself) that are used to register three ECG vectors. The primary vector is recorded between the proximal ring of the lead (the so-called B sense ring) and the can. The secondary vector is recorded between the distal ring on the lead (the so-called A sense ring) and the can. And the third vector — alternate — is recorded between the distal and proximal sensing rings on the lead (see Fig. 1).

Once the device is implanted, only one of those three vectors can be selected for permanent use and detection, which is an obvious limitation of the system. To check the chances of the system for appropriate detection, a preoperative analysis has to be undertaken to verify the eligibility of the ECG signals, that is the morphology of the QRS complex in simulated sensing vectors. That procedure was historically performed with a screening template applied to a printout of modified surface ECG leads, but nowadays it is routinely executed with an automated screening tool, being a proprietary software for ECG analysis implemented in the programmer of the device manufacturer (Boston Scientific). For that purpose, the standard adhesive ECG patches have to be placed on the skin over predicted locations of the device sensing poles (two on the course of the lead and one over the pocket), based on the observation and palpation of anatomical landmarks on the chest, aided with fluoroscopic verification (if necessary). Then the ECG leads of the programmer have to be attached according to the on-screen instructions: Left Leg (LL) lead — laterally on the chest, along the mid-axillary line in the fifth intercostal space, to match the future location of the pocket and the device can; Left Arm (LA) lead — 1 cm left from the xyphoid process of the sternum to match the future location of the lower (proximal) sensing ring on the lead; Right Arm (RA) lead — 14 cm above the previous one, to match the predicted location of the distal sensing ring of the lead. The software performs an analysis of ECG strips simulating three sensing vectors and decides whether they are “OK” or they “FAIL” the screening procedure. The result is displayed on-screen and can be saved for printout. The ECG screening is typically performed in two body positions — supine and upright. According to system specifications, at least one vector should be “OK” in all analyzed body positions. In our clinical practice, we qualify patients with at least two passing vectors (the same in both supine and standing body positions) to ensure any room for error, if vectors after implantation differ from preoperative screening. Some experts accept only one passing vector, but then it should be checked in more body positions (for example lying on both sides in addition to supine and upright). If screening is negative, alternative lead location (right parasternal) may be considered [11, 12, 13, 14, 15]. In specific situations (congenital heart disease, dextrocardia) different positions of the system (e.g., reversed right-to-left) have been described [16, 17, 18, 19], but if such a case is planned, the ECG screening should incorporate fluoroscopy to ensure the correct relation of S-ICD system components to the cardiac silhouette and location of the heart within the chest in relation to anatomical landmarks [20]. On the other hand, the ECG screening may be dynamic, and repeated recordings are likely to give contradictory results [21]. In some specific clinical conditions [arrhythmogenic right ventricular cardiomyopathy (ARVC), hypertrophic cardiomyopathy (HCM), Brugada syndrome (BS) and congenital heart disease (CHD)] the percentage of failed screening may be higher than in other groups of patients [22, 23, 24, 25, 26, 27, 28], and may require special considerations (like repeated screening during exercise in ARVC or during drug challenge in BS) [29, 30]. To make the issue even more complex, in some circumstances the change of QRS morphology may lead to misinterpretation of signals by S-ICD despite prior positive screening. That QRS morphology change may be due to pocket hematoma, cardiac ischemia, exercise, rate-dependent or transient bundle branch block, alcohol septal ablation in HCM, ST segment changes (in BS, electrolyte disturbances or after dialysis), cardiac pacing or lead migration [31, 32, 33, 34, 35, 36, 37, 38].

Meticulous screening and qualification for S-ICD are of paramount importance, as inappropriate sensing and shocks (IAS) remain one of the main ICD-related adverse events. In the early years of development of the S-ICD system, IAS affected a significant percentage of patients, with T-wave oversensing being the most frequent reason. Those observations encouraged the manufacturer to modify the sensing algorithm, and as a result the Smart Pass filter was introduced. The filter reduces the amplitude of lower frequency signals (T-waves), selectively letting higher frequencies pass through (so that higher frequency signals such as R-waves and ventricular arrhythmias are not attenuated). The Smart Pass filter reduced the rate of IAS [39, 40], but they were not completely eliminated. In recently reported series, the IAS rate remained substantial, although not higher than in TV-ICDs, and it was not related to any clinical factors (such as age, body mass index, structural heart disease, left ventricular ejection fraction or sensing vector) apart from ARVC (where it was higher) [40, 41, 42].

Once the decision was made to implant an S-ICD system, appropriate implantation techniques have to be selected. There are several key technical issues to be considered before and during implantation, including the type of anesthesia, the number of skin incisions for lead tunneling and the location and type of the device pocket.

## 4. Anesthesia

The procedure may be performed in general, regional [43, 44] or local anesthesia [45]. In the center in Gdansk we typically implant S-ICD systems in general anesthesia, with laryngeal mask for mechanical ventilation, unless decided otherwise by an anesthesiologist. Performing implantations in general anesthesia allows to proceed directly to defibrillation testing (DFT). Moreover, if any problem arises that might require revision or repositioning of the system (although it is very rare with correct implantation techniques), it can be performed easily. Therefore the general anesthesia is usually a first choice for centers starting S-ICD implantation. Other centers prefer to use regional anesthesia in the form of a fascial plane block [46, 47, 48, 49]. That technique may be performed outside the operating room (in a preparatory room or even as a bedside technique), before the actual onset of the procedure, and therefore may increase the operational volume and speed up the workflow. But it requires specific skills from the anesthesiologist, and preferably the use of ultrasound imaging [50]. Local anesthesia is less commonly used, as the area of skin and subcutaneous tissue needing anesthetic infiltration is relatively large, and therefore a dose of local anesthetics and number of puncture sites limit the use of that method. According to available data, general anesthesia is the most prevalent method [51] but centers evolve over time and those rates may change.

## 5. The Lead

**Fig. 2.**
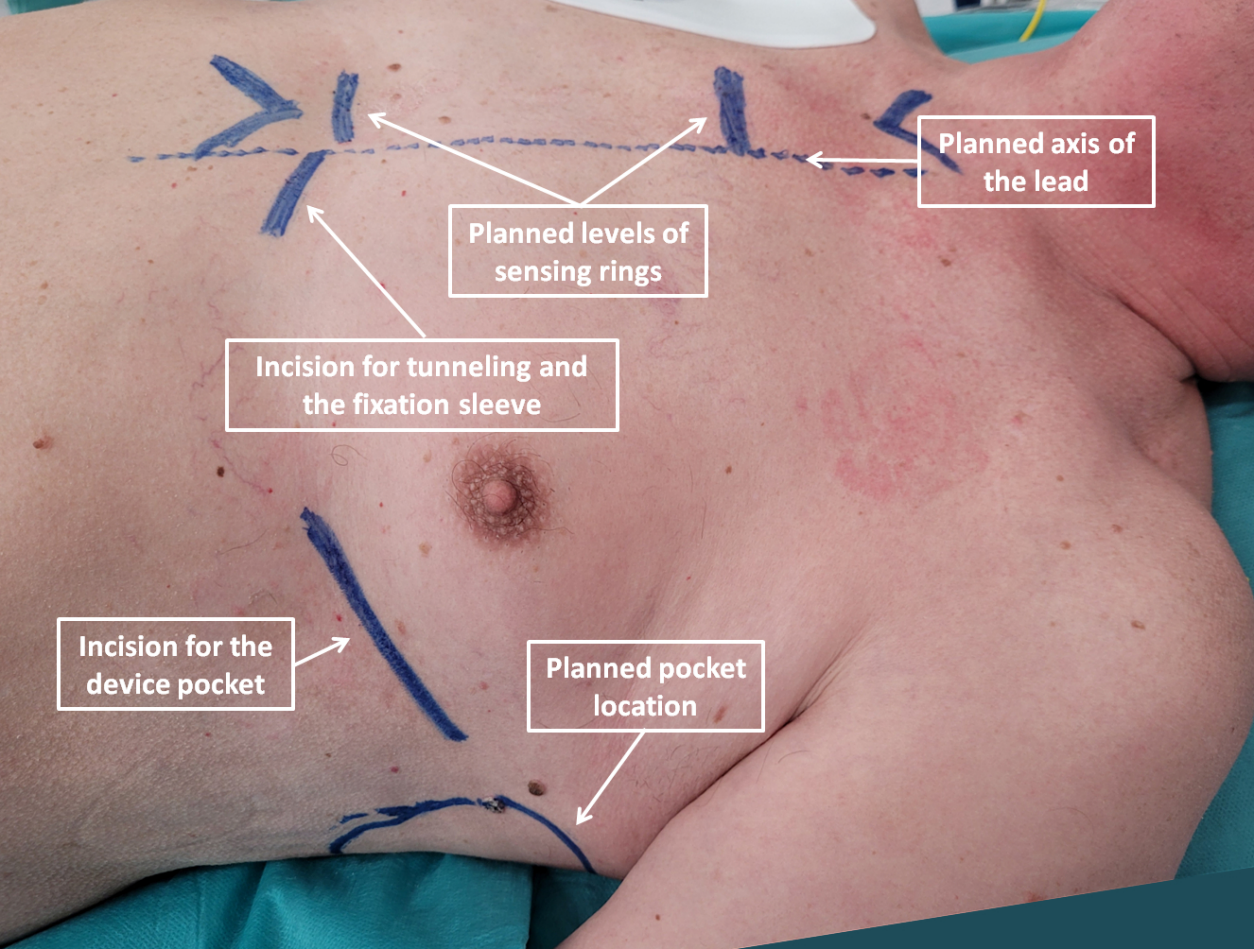
**Anatomical landmarks and planned incisions marked on patient’s skin**.

As mentioned above, preoperative planning should define the future locations of the system components. The anatomical landmarks may be marked on patient’s skin before implantation, and a dummy system may be used together with fluoroscopy to plan the position of the system and skin incisions. Fluoroscopy is especially useful in case of atypical anatomy of the chest and/or the heart. An example of preoperative planning and marking of the anatomical landmarks and skin incisions is presented in Fig. 2.

**Fig. 3.**
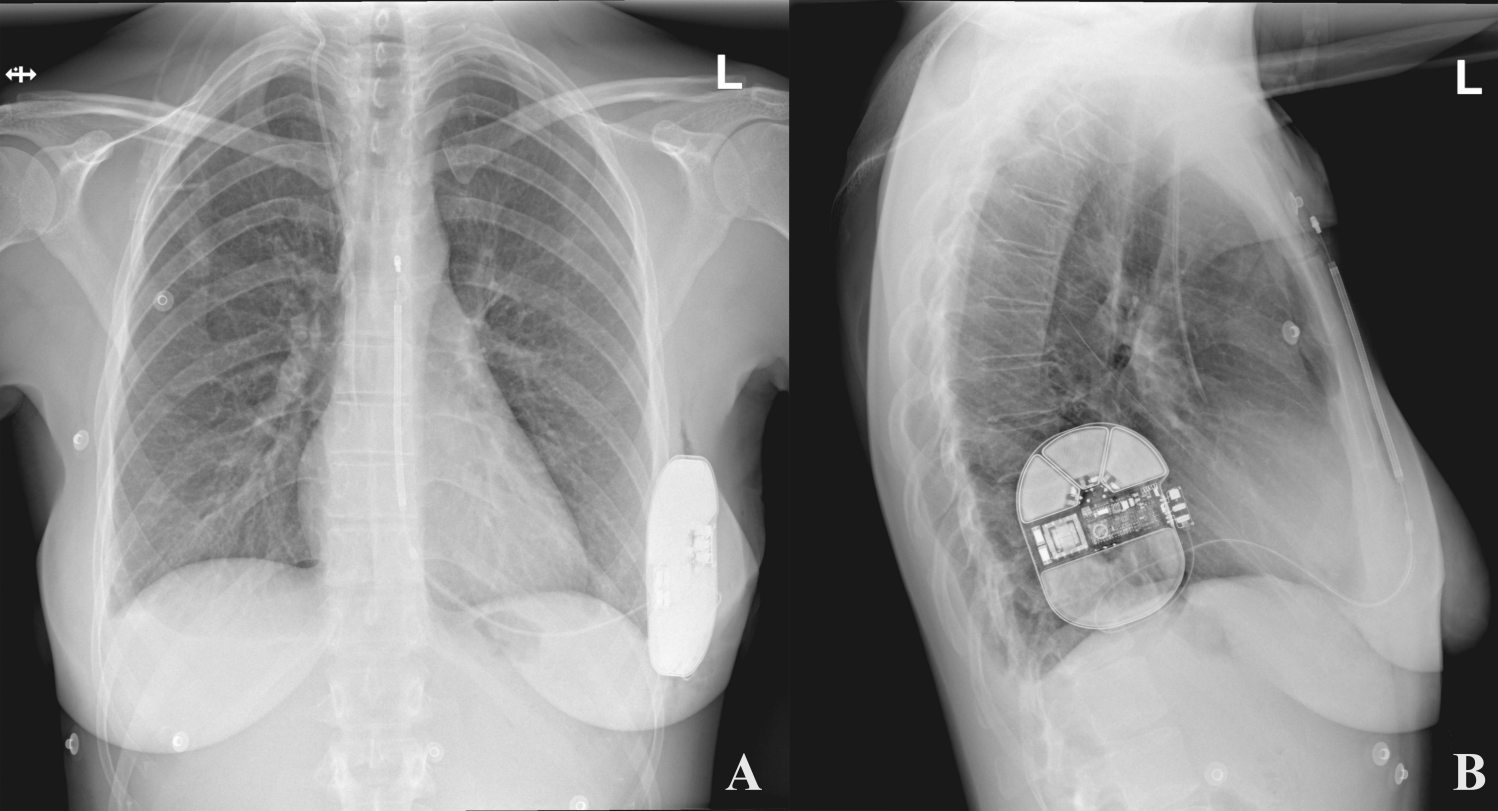
**An exemplary postoperative chest x-ray presenting an appropriate position of the S-ICD system**. (A) Postero-anterior view. (B) Lateral view. Note the posterior location of the can, which is typically obtained with the intermuscular pocket. S-ICD, subcutaneous implantable cardioverter-defibrillator.

The lead should be implanted parallel to the sternum, preferably along the left sternal margin. To obtain the best system impedance and optimal position for conversion of ventricular arrhythmias, the lead should be placed as deep as possible (close to the sternum and the pectoralis fascia), to leave the subcutaneous tissue and fat above. The final position of the lead should also be on the height of the cardiac silhouette. An example of correct lead placement is presented in Fig. 3.

The S-ICD system implantation may be performed using a so-called two- or three-incision technique [52, 53]. One skin incision for the pocket is a must, so is the second one at the xyphoid region for lead tunneling and lead sleeve fixation. Therefore the decision has to be made only whether to perform the third incision for lead tunelling along the sternum at the upper parasternal region (leftwards from the sternomanubrial junction). The lead is tunneled from the pocket to the xyphoid region using the long stylet and a long peel-away sheath provided in the lead tunneling set. Then sutures for fixation of the suture sleeve (which in the current design of the lead is integrated with the lead body and cannot slide) have to be planned and placed in the xyphoid region. If the third incision was made, a bare tunneling stylet can be used to perform the tunnel along the left margin of the sternum, and the lead can be then dragged through the tunnel using suture strings (left at the cranial end of the tunnel after stylet removal). If the third incision is not performed, the lead has to be placed using the short peel-away sheath delivered over the short tunneling stylet from the tunneling set. In our experience, it is of uttermost importance to predefine the straight line for tunneling and to avoid rocking to the sides with a stylet during ist course along the sternum, as it might subsequently produce the twisting course of the lead. If the tunneling line is for any reason more lateral than the parasternal line, then the lead may run through the attachment of the pectoralis major muscle, and deviate slightly from the straight line after sheath removal. The stylet is then removed, and the lead is inserted into the sheath. In the following step the sheath has to be peeled away gently, so as not to dislocate or remove the lead along with the sheath. Holding the lead at the fixation sleeve region (for example with anatomical forceps) may aid against dislocation, but a second pair of hands (assisting physician or nurse) may facilitate that maneuver. Then the final position of the lead has to be attained, using pre-specified anatomical landmarks and previously prepared sutures. Before closing the para-xyphoid incision, we typically pour some saline into the wound using a syringe (but not under pressure into the lead tunnel), and gently push the skin down over the lead tunnel from the cranial end towards the incision site, to push away any excessive air (air bubbles are typically seen passing through the saline pond). Care has to be taken during that maneuver to avoid manual dislocation of the lead. When closing the wound, the sutures should not be pulled up (away from the patients body), as it might facilitate air entrapment around the lead. If for any reason the final lead position is not acceptable, or there was an early dislocation, we would advise conversion to the three-incision technique, as repetitive tunneling weakens the surrounding tissue and may increase the risk of subsequent lead dislocation. In such a case, the third incision allows for secure fixation with a suture at the distal end of the lead. As mentioned before, in case of any doubt concerning the final placement of the system components, fluoroscopy may and should be used for verification.

The initial choice between two- or three-incision technique is based on several considerations. The clinical outcomes seem to be comparable between those two techniques in terms of the rates of infection, electrode migration, inappropriate shocks and first shock efficacy, but the two-incison technique is more time-efficient [54]. There is also a report available of a single-incision technique [55], but that approach has not yet gained wider acceptance. The two-incision procedure typically produces a better cosmetic effect than the three-incision one, as it allows to avoid the upper wound at the sternomanubrial junction (the most exposed and visible). The remaining two wounds (i.e., the only two if the two-incision technique is applied) in female patients can be easily hidden beneath underclothes and therefore are more cosmetically acceptable. Patients with excessive subcutaneous tissue could benefit from the 3-incision technique, as it allows for more stable fixation if a thick layer of fatty tissue is present, although in one report the complication rate in obese patients was not significantly increased [56].

**Fig. 4.**
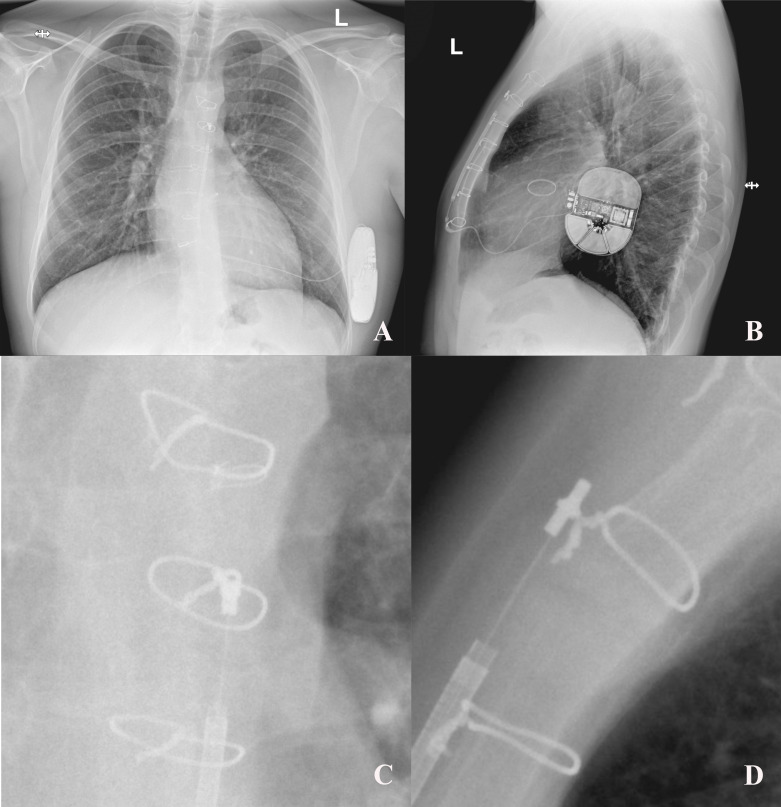
**Example of sternal wires touching the S-ICD lead**. The patient (implanted elsewhere) presented to our center with inadequate interventions due to noise oversensing. (A) Postero-aterior view. (B) Lateral view. (C) Postero-anterior view, close-up of the lead tip in contact with a sternal wire. (D) Lateral view, close-up of the lead tip in contact with a sternal wire. S-ICD, subcutaneous implantable cardioverter-defibrillator.

Another lead-related issue that should not be underestimated is the interaction with sternal wires in patients after cardiac surgery with sternotomy. Direct contact of sensing points (lead tip or proximal ring) results in oversensing of noise and inadequate interventions [36]. Therefore the lead tunnel should be more lateral to step aside from the sternal wires in those patients, and intraprocedural fluoroscopy may help to acsertain the lack of contact between sensing rings and the sternal wires. The device manual does not specify the minimal distance, but definitely any direct contact should be avoided. For the same reason, if sternotomy is planned or likely to occur, the lead course should allow for that. Cardiac surgeons should be aware of the potential presence of a parasternal lead, especially because S-ICDs are still less common than TV-ICDs and in some centers physicians other than electrophysiologists may be less acquainted with that type of device. If they are not aware of the lead presence, it may end in contact or even entrapped within sternal wires after cardiac surgery. That in turn may lead to inappropriate sensing and shocks, as well as render the lead irremovable. An example of wires touching the lead is presented in Fig. 4. The clinical outcomes of post-sternotomy patients implanted with an S-ICD are similar to the rest of S-ICD recipients [57].

Lead tunneling should be performed with both confidence and caution, as the inadvertently deviated course of the tunneling tool may lead to complications, for example if it passes under the sternum through the intercostal space. Such a trajectory might result in damage to the lung or — even worse — the heart [58, 59]. On the other hand, the deliberately substernal location of an S-ICD lead was reported as a measure to overcome unsuccessful DFT in S-ICD recipients [60, 61]. Such a technique definitely cannot do without a cardiac surgeon at least on stand-by or in the operating team.

The S-ICD lead has a robust structure, as it does not have a central lumen for a stylet. The overall lead fracture rate remains low (around 0.3%) [62], especially when contrasted with the failure rate of transvenous ICD leads.

A relatively new problem concerning the S-ICD lead is the so called “sense-B-noise issue”. Several patients were described to have a the possible system and lead malfunction manifesting as electrical noise in sensing vectors involving the lower sensing ring (i.e., primary and alternate vectors). It led to inappropriate shocks, and required at least reprogramming to a secondary vector or total system replacement, even in case of no apparent lead damage [63]. The nature of that phenomenon remains unclear and is under investigation.

## 6. The Pocket

In a typical setting, the device can is placed in the pocket located on the left lateral side of the chest. Preoperative planning with a dummy system allows for correct final position of its components in relation to the cardiac silhouette and individual anatomy of a patient. In most cases, the final position of the can is on or posterior to the left midaxillary line, at the level of 5th and 6th intercostal space. The precise location of the pocket underwent evolution with growing experience in S-ICD implantation. Initially, the pocket was created subcutaneously (mimicking the technique used before for TV-ICDs, but now in another location). Evolution of the implantation technique due to pocket-related complications led to a wider acceptance of the intermuscular technique, usually combined with the two-incision access [64, 65, 66, 67]. Subcutaneous location of the pocket, due to the size of the device, may lead to excessive skin tension, and subsequently — skin erosion and device extrusion [68]. Local pocket complications of S-ICDs, as opposed to TV-ICDs, may undergo attempts of conservative treatment with a high rate of success, especially in non-infective cases. The same applies to lead-related surgical problems [69, 70]. In our early experience we successfully treated a patient implanted elsewhere using a subcutaneous pocket, and presenting to our center with a threatening pocket erosion. Covering the device with a displaced latissimus dorsi muscle resulted in complete healing of the pocket [71]. Since then, the wide acceptance and use of the intermuscular technique reduced the surgical complications to a consistently low level [72]. In a recent propensity-matched comparison, intermuscular pocket was found to be superior to a subcutaneous one in reducing device-related complications and inappropriate shocks [73].

The surgical access for creating the pocket is typically achieved with an incision along the inframammary crease, adhering to the Langer’s lines of skin tension (see Fig. 2). Then the subcutaneous pocket would have typically been performed by dissection of the subcutaneous tissue over the muscular plane of the lateral chest, and the device secured to the facia of the serratus anterior muscle. Creating the intermuscular pocket requires dissection down to the muscular fascia, and then dorsally, until the front edge of the latissimus dorsi muscle is found. The crucial step is not to overlook the margin of that muscle. Then the latissimus dorsi muscle can be in most cases separated from the serratus anterior muscle with blunt dissection. The pocket is created between those two muscles, with the latissimus dorsi at least partially covering the device can. As mentioned before, that approach allows for the optimal position of the can, as judged by two factors: (1) the can is deep under the skin and close to the chest wall, i.e., with low or no fat tissue layer between the can and the rib cage, and (2) a relatively dorsal position of the can. Those two factors were found to increase the rate of successful DFT [74, 75]. The third pocket variant is a submuscular pocket, under the serratus anterior muscle. That type of pocket is used least frequently, although it may be applied if needed in specific cases [76].

There are several important considerations that have to be remembered during pocket creation and closure. First, the device has to be firmly secured to the wall of the chest (preferably to the muscular fascia), to avoid dislocation of the can, as it might change sensing vectors and lead to inappropriate interventions [77]. Second, a meticulous surgical technique and hemostasis are needed to avoid pocket hematoma, as it might affect sensing (the can is used for primary and secondary vectors) and efficacy of defibrillation. Anticoagulation and antiplatelet therapy are associated with an increased risk of hematoma in case of S-ICD implantation. Interruption of anticoagulation without bridging should be considered, if possible [78, 79]. And third, the pocket has to be the right size. Too snug a pocket may increase the risk of skin erosion. Too loose — may facilitate encapsulation of air around the device can. The remarks considering air entrapment around the lead above apply also to the pocket. Filling the pocket with saline before closure may expell excessive air. Gentle pressure applied to the skin over the pocket during closure may be another possible measure to push air away. And, last but not least, when connecting the lead to the can, the screwdriver has to be inserted into the screw through the sealing plug to allow for free outflow of air from the connector during lead insertion. Failure to obey the last rule may also lead to noise sensing and inappropriate interventions. Air entrappment around various components of the system has been reported multiple times [80, 81, 82, 83]. It is not associated with any specific technique, and — unfortunately — cannot be eliminated completely [84]. The air is typically resorbed within several days. According to our experience, it may be reasonable to switch off the device after implantation and DFT (if performed), and switch it on again the next day, when the presence of an excessive amount of air has been excluded with a routine chest x-ray.

## 7. Defibrillation Testing

The manufacturer recommends DFT after implantation of the S-ICD system, unless contraindicated. In case of TV-ICDs, DFT has been largely abandoned, especially after the non-inferiority of such a scenario was confirmed in the cardioverter defibrillator implantation without induction of ventricular fibrillation: a single-blind, non-inferiority, randomised controlled trial (SIMPLE trial) [85]. The results of that study cannot and should not be translated into S-ICD routine. But current practice shows that in some patient series the DFT is skipped in many cases of first-time implantation and in even more cases of device exchange [86]. More and more attempts are made to completely eliminate DFT for S-ICDs, as described below.

DFT should be typically performed after implantation of the system and wound closure. General anesthesia is continued (if it was used for implantation) or started only for that step (if regional techniques were used for implantation). Ventricular fibrillation (VF) is induced by the in-built protocol of the device with an induction impulse. If appropriate sensing occurs, the device detects VF, loads the capacitor and delivers a programmed shock. The maximum energy output of the device is 80 J, and the value of 65 J is typically programmed for DFT to maintain a 15 J safety margin, although some reports prove that with correct implantation even lower values (20-40-50 J) can be efficient [87, 88, 89]. If patient’s arm was abducted for implantation, it should adducted back for DFT to avoid trauma during forceful muscle contraction occurring at VF induction [90, 91]. A successful 65 J shock with correct impedance (40–140 Ohm) ends the test. If that is not the case, the reversed polarity should be tested, provided that the impedance is in the acceptable range. If the impedance is out of range and/or the shock was not successful, the position of the system should be re-checked and corrected, if possible [92]. Reference impedance values given in the device manual are between 25 and 110 Ohm, values below 25 or over 200 Ohm trigger patient’s alert.

Clinical experience and digital modeling suggested that there are certain determinants increasing the probability of VF conversion, namely the posterior position of the generator, and as little fat as possible between the lead coil and the can, and respective muscular surfaces underneath [75, 93]. Following that, an S-ICD specific score (PRAETORIAN score) was developed to correlate the final position of the system with the probability of a successful DFT [74]. The score is based on the analysis of post-operative chest x-rays (dual view, posteroanterior (PA) and lateral), used to assess the location of the lead in relation to the sternum, as well as the position of the can in the postero-anterior aspect and in relation to the chest wall. The final value is adjusted for patient’s body mass index. The final score reflects numerically the “correctness” of the position of the system and the amount of fat tissue beneath its components. The value below 90 indicates a low risk, and above 150 - a high risk of DFT failure, respectively. A score was validated on an independent cohort of S-ICD recipients [94, 95], and it holds true even in obese patients [96]. Intraoperative calculation of the score was proved feasible [97]. But appropriate determination of the score is to some extent subjective, and may necessitate some form of training [98].

In patients with contraindications for a DFT, a 10 J test shock with no VF induction is considered a surrogate of DFT, as it allows for true high-voltage impedance measurement (and not its low-voltage approximation). That method cannot confirm appropriate VF sensing and detection, and the value of impedance measurement only is disputable [99, 100], as it may vary with the patient’s body composition and built, chest fluid content (e.g., pleural effusion) and device location. There are several reports available suggesting that DFT testing may be safely waived in S-ICD patients [101, 102], but more conclusive data are awaited from the PRAETORIAN-DFT trial, which was designed to investigate the possibility of substituting the DFT with PRAETORIAN score calculation only [103].

## 8. Coexistence with Other Devices

S-ICD systems, relying on the analysis of ECG-like farfield signals (as opposed to TV-ICDs, that analyze bipolar local intracardiac electrograms), are more prone to interference with any other devices and circumstances generating noise or alteration of the sensing signal. As mentioned before, in the early years of S-ICD therapy, T-wave oversensing leading to inappropriate interventions was a serious issue (reaching 7–15% of patients) [104, 105, 106, 107]. Later on, when the in-built filters and sensing algorithms were refined [108, 109], the incidence of inappropriate shocks decreased to the level comparable to TV-ICDs [5, 110]. Nonetheless, possible interaction with other devices remains a serious issue. Any implantable device containing magnets (like for example some insulin pumps) may theoretically induce electromagnetic noise sensed by S-ICD. More concern is raised by other devices, that operate delivering electrical impulses within the patient’s body.

Left ventricular assist devices were reported to cause electromagnetic interference and inappropriate shocks in S-ICD patients [111, 112, 113]. According to a recent systematic review, interference was recorded in the primary and secondary vectors in the majority of cases, and reprogramming to the alternate vector could potentially solve sensing issues [114].

Other devices that may interact with S-ICD include pacing systems for deep brain stimulation, cardiac contractility modulation systems, baroreceptor stimulators and vagus nerve stimulators. All of those devices were described in the context of possible interference [115, 116]. Some of them can be applied in selected patients [117, 118, 119, 120], but a case-by-case analysis and thorough investigation of any possible cross-talk is crucial in those patients, and should involve in-depth analysis with programmers for all implanted devices and various possible current outputs of the devices.

## 9. Coexistence with Transvenous Cardioverters-Defibrillators and Pacemakers

Typical indications for S-ICD include a history of infection of a transvenous system and its extraction. Implantation of S-ICDs in such a clinical setting was found to be feasible and safe in terms of further risk of infection [121, 122, 123, 124]. The need for resynchronization therapy, ATP and permanent pacing has to be considered before a decision is made to folow the S-ICD path. If non-infective indications for discontinuation of TV-ICD therapy occur (e.g., repetitive lead failure, failed extraction, no vascular access), a decision may be made to abandon a transvenous system in its location or the transvenous lead alone, and implant an S-ICD system in addition [125].

Some S-ICD patients may develop indications for permanent pacing or resynchronization therapy even if they were not present at the initial device choice. In such a situation, a decision to extract the S-ICD and convert to a transvenous pacemaker or ICD can be made. Alternatively, a combination therapy with a pacemaker and S-ICD may be attempted. The former idea is straightforward, but the latter has to be discussed in further detail. Another scenario of possible co-therapy with an S-ICD and a pacemaker is when a patient with pre-existing pacemaker requires an ICD, and conversion to a TV-ICD is not an option (e.g., in case of epicardial systems or leadless pacing). Of course those clinical settings may have numerous variants, but we will try to detail some of the possible patterns.

Many cases of successful S-ICD implantation were described in patients with pre-existing pacemakers [126, 127], but careful screening of paced rhythm is needed. Some pacing sites and modalities (septal right ventricle pacing, biventricular pacing, His-bundle pacing) may produce more S-ICD eligible QRS morphologies than others (apical right ventricle pacing) [128, 129, 130, 131]. Epicardial pacing systems, both conventional [36, 132] and resynchronization therapy ones [133], were also successfully co-implanted with S-ICDs. In all of the above situations, the decision to add an S-ICD to a pacemaker may be made after positive screening. If screening is negative, other solutions have to be applied. A more complex situation occurs, when a pacemaker needs to be added to a pre-existing S-ICD. In such a case, evaluation of the QRS morphology of the paced rhythm and its sensing by an S-ICD can be performed only after pacemaker implantation. In case of failed screening, the pacemaker cannot be left in place. To add even more complexity, if the paced rhythm interchanges with the intrinsic one, positive screening and appropriate sensing may be even less achievable, because then we need a positive result for both types of rhythm in one and the same vector in both body positions [134].

Epicardial pacing systems seemed to be a reasonable solution for S-ICD recipients who developed indications for pacing, because they allowed overcomig the pacing need without entering the cardiovascular system. But another option emerged in the recent years than can be used instead – leadless pacemakers (LP). LPs undergo fast epithelialization and are therefore less prone to infection. As such, they are sometimes used in case of lead-dependent infective endocarditis in pacing-dependent patients [135]. For the same reason they started to be considered as a good solution for S-ICD patients in need for pacing (being the lesser evil inside the heart compared to traditional transvenous pacemakers). Since the first report of such a combined system [136], several patients were reported worldwide to have good outcomes of such a therapy [137, 138, 139, 140].

Concomitantly with the above reports, the manufacturer of S-ICD undertook research to develop the proprietary combined S-ICD and LP system [141, 142]. That work is ongoing, and the system has been already implanted in humans [143], but the date of market release — being much awaited — remains unknown.

## 10. Removal of the S-ICD System

The need for S-ICD removal may occur in several circumstances. First, if indications develop over time for permanent cardiac pacing, ATP or cardiac resynchronization therapy, although the cumulative risk of conversion to TV-ICD is low (2.7% in a recent report) [144]. Second, in case of infection or erosion of the device that cannot be treated conservatively [70], although the rate of pocket and lead-related complications is lower for S-ICDs than TV-ICDs [5, 145]. And third, if the potentially reversible cause for S-ICD cured or was eliminated in rare cases (improvement of heart failure due to toxic cardiomyopathy or myocarditis, heart transplant).

Irrespective of the reason for S-ICD explantation, the procedure itself may be not as straightforward as expected. Although extraction of an S-ICD system is obviously easier than of a TV-ICD one, due to the lack of contact with the cardiovascular system, some tools and techniques used in transvenous lead extraction may also be needed. Simple traction is not always successful, additional skin incisions may be needed, and the use of mechanical sheaths and even rotational mechanical sheaths has been reported [146, 147, 148, 149, 150]. As mentioned before, lead entrapment within sternal wires should be excluded in patients after sternotomy before S-ICD removal is attempted.

## 11. Conclusions

S-ICD system is a valuable addition to our antiarrhythmic armamentarium. It may successfully replace a TV-ICD in many patient populations, reducing the risk of vascular and lead-related problems. To keep the rate of S-ICD specific complications low, and to replicate the results of clinical studies in everyday practice, an implanting physician may choose to apply the two-incision technique with intermuscular pocket. Future development of the system and integration with leadless pacing may broaden indications and increase the target population for that system.

## Abbreviations

ARVC, arrhythmogenic right ventricular cardiomyopathy; ATP, antitachycardia pacing; BMI, body mass index; BS, Brugada syndrome; CHD, congenital heart disease; DFT, defibrillation testing; HCM, hypertrophic cardiomyopathy; IAS, inappropriate shock; ICD, implantable cardioverter-defibrillator; LP, leadless pacemaker; SCD, sudden cardiac death; S-ICD, subcutaneous implantable cardioverter-defibrillator; TV-ICD, transvenous implantable cardioverter-defibrillator; VF, ventricular fibrillation; VT, ventricular tachycardia.
